# Inhibition of G Protein-Activated Inwardly Rectifying K^+^ Channels by Phencyclidine

**DOI:** 10.2174/157015911795017407

**Published:** 2011-03

**Authors:** Toru Kobayashi, Daisuke Nishizawa, Kazutaka Ikeda

**Affiliations:** 1 Division of Psychobiology, Tokyo Institute of Psychiatry, 2-1-8 Kamikitazawa, Setagaya-ku, Tokyo 156-8585, Japan; 2 Department of Project Programs, Center for Bioresource-based Researches, Brain Research Institute, Niigata University, 1-757 Asahimachi, Chuo-ku, Niigata, Niigata 951-8585, Japan

**Keywords:** Phencyclidine, GIRK channel, intoxication, Kir channel, *Xenopus* oocyte.

## Abstract

Addictive drugs, such as opioids, ethanol, cocaine, amphetamine, and phencyclidine (PCP), affect many functions of the nervous system and peripheral organs, resulting in severe health problems. G protein-activated inwardly rectifying K^+^ (GIRK, Kir3) channels play an important role in regulating neuronal excitability through activation of various Gi/o protein-coupled receptors including opioid and CB_1_ cannabinoid receptors. Furthermore, the channels are directly activated by ethanol and inhibited by cocaine at toxic levels, but not affected by methylphenidate, methamphetamine, and 3,4-methylenedioxymethamphetamine (MDMA) at toxic levels. The primary pharmacological action of PCP is blockade of *N*-methyl-D-aspartate (NMDA) receptor channels that are associated with its psychotomimetic effects. PCP also interacts with several receptors and channels at relatively high concentrations. However, the molecular mechanisms underlying the various effects of PCP remain to be clarified. Here, we investigated the effects of PCP on GIRK channels using the *Xenopus* oocyte expression system. PCP weakly but significantly inhibited GIRK channels at micromolar concentrations, but not Kir1.1 and Kir2.1 channels. The PCP concentrations effective in inhibiting GIRK channels overlap clinically relevant brain concentrations in severe intoxication. The results suggest that partial inhibition of GIRK channels by PCP may contribute to some of the toxic effects after overdose.

## INTRODUCTION

G protein-activated inwardly rectifying K^+^ (GIRK) channels (also known as Kir3 channels) are members of a family of inwardly rectifying K^+^ (Kir) channels that includes seven subfamilies [1]. Four GIRK channel subunits have been identified in mammals [1]. Neuronal GIRK channels are predominantly heteromultimers composed of GIRK1 and GIRK2 subunits in most brain regions or homomultimers composed of GIRK2 subunits in the substantia nigra, whereas atrial GIRK channels are heteromultimers composed of GIRK1 and GIRK4 subunits [2]. GIRK channels play an important role in the inhibitory regulation of neuronal excitability in most brain regions and heart rate through activation of various Gi/o protein-coupled receptors, such as opioid, CB_1_ cannabinoid, and D_2_ dopamine receptors [2]. Furthermore, the channels are modulated by various psychoactive agents, such as ethanol, antipsychotics, antidepressants, anesthetics, and hormones [2-11]. Recently, we demonstrated that cocaine at toxic levels inhibited GIRK channels expressed in *Xenopus* oocytes. In contrast, methylphenidate, methamphetamine, and 3,4-methylenedioxymethamphetamine (MDMA) at toxic levels had little effect on GIRK channels, although these drugs at higher concentrations inhibited the channels to a lesser extent than cocaine [12].

Phencyclidine (PCP) has been used as a drug of abuse, although it was originally developed as a general anesthetic in the 1950s [13, 14]. The primary pharmacological action of PCP is blockade of *N*-methyl-D-aspartate (NMDA) receptor channels that are associated with its psychotomimetic effects [13, 14]. PCP at relatively high concentrations interacts with several receptors and channels, namely, σ, µ-opioid, nicotinic- and muscarinic-acetylcholine receptors, voltage-gated K^+^, Na^+^ and Ca^2+^ channels, and adenosine triphosphate (ATP)-sensitive K^+^ channels [14, 15, 16]. However, the molecular mechanisms underlying the various effects of PCP have not yet been sufficiently clarified. In the present study, we investigated the effects of PCP on GIRK channels and other Kir channels using the *Xenopus* oocyte expression system.

## METHODS

For *Xenopus* oocyte experiments [4, 5], *Xenopus* *laevis* oocytes were injected with mRNA for GIRK1/GIRK2 or GIRK1/GIRK4 combinations, GIRK2, Kir1.1, or Kir2.1. The oocytes were incubated at 19°C in Barth's solution and defolliculated after collagenase treatment. Whole-cell currents of the oocytes were recorded with a conventional two-electrode voltage clamp. Oocytes were superfused with a high-potassium solution containing 96 mM K^+^. The membrane potential was held at -70 mV. The values obtained are expressed as mean ± SEM, with *n* indicating the number of oocytes tested. PCP was generously provided by Shionogi Pharmaceutical Co. Ltd. (Osaka, Japan).

## RESULTS

In *Xenopus* oocytes injected with GIRK1 and GIRK2 mRNA, PCP reversibly reduced basal GIRK inward currents (Fig. (**1A**)). Similar results were observed in oocytes injected with either GIRK1 and GIRK4 mRNA or GIRK2 mRNA. However, in oocytes expressing either Kir1.1 or Kir2.1 channels, PCP caused no significant response even at 100 µM (less than 5 nA, *n* = 4, Fig. (**1A**)). Additionally, in uninjected oocytes, 100 µM PCP and 3 mM Ba^2+^, a Kir channel blocker, caused no significant response (Fig. (**1A**)). The results suggest that PCP inhibits GIRK channels. The inhibition by PCP was concentration-dependent (*n* = 4, Fig. (**1B**)).

## DISCUSSION

We demonstrated that PCP at micromolar concentrations inhibited brain-type GIRK1/2 and GIRK2 channels and atrial-type GIRK1/4 channels expressed in *Xenopus* oocytes. At 100 ìM or less, the inhibitory effects of PCP were more potent than those of cocaine, methylphenidate, methamphetamine, and MDMA (Fig. (**2**)). In other Kir channel subfamilies, Kir1.1 and Kir2.1 channels were insensitive to these psychostimulants [12] and PCP, whereas PCP inhibited cardiac ATP-sensitive K^+^ channels, which comprise four pore-forming Kir6 subunits and four regulatory sulfonylurea receptor subunits, with an IC50 value of approximately 20 µM [16]. Further studies using GIRK/Kir1.1 and GIRK/Kir2.1 chimeric channels and mutant GIRK channels may clarify the critical sites mediating the effects of PCP on GIRK channels.

The use of PCP as a drug of abuse is an important medical problem. Serum PCP concentrations after overdose were reported to reach up to approximately 10 µM in some postmortem cases, up to 45 µM in one massive overdose case [17], and up to approximately 3.3 µM in some nonfatal cases [18], although serum concentrations in most intoxication cases ranged widely from nanomolar to low micromolar concentrations [17, 18]. Furthermore, the concentrations in the brain were reported to be from 3 to 14-fold higher than those in serum [19]. Additionally, because the early phase elimination rate after administration of PCP is relatively high [19], the peak concentrations would be higher than the concentrations measured. Therefore, the PCP concentrations effective in inhibiting GIRK channels overlap the clinically relevant brain concentrations in severe intoxication or fatal cases.

PCP concentrations in severe intoxication cases are associated with coma, seizures, muscle rigidity, and respiratory arrest [20]. GIRK2 knockout mice show spontaneous seizures and are more susceptible to seizures induced by pentylenetetrazol than wild type mice [21]. Furthermore, GIRK inhibitors can depolarize the membrane potential and induce action potentials [22]. Because GIRK channels play an important role in regulating cell excitability, even partial inhibition of neuronal GIRK channels by PCP might contribute to the incidence of seizures and some of neuropsychiatric complications observed in severe or fatal cases after overdose. GIRK channels may be considered as important molecules mediating the effects of PCP, cocaine, opioids, cannabinoids, and ethanol among addictive drugs.

## Figures and Tables

**Fig. (1):**
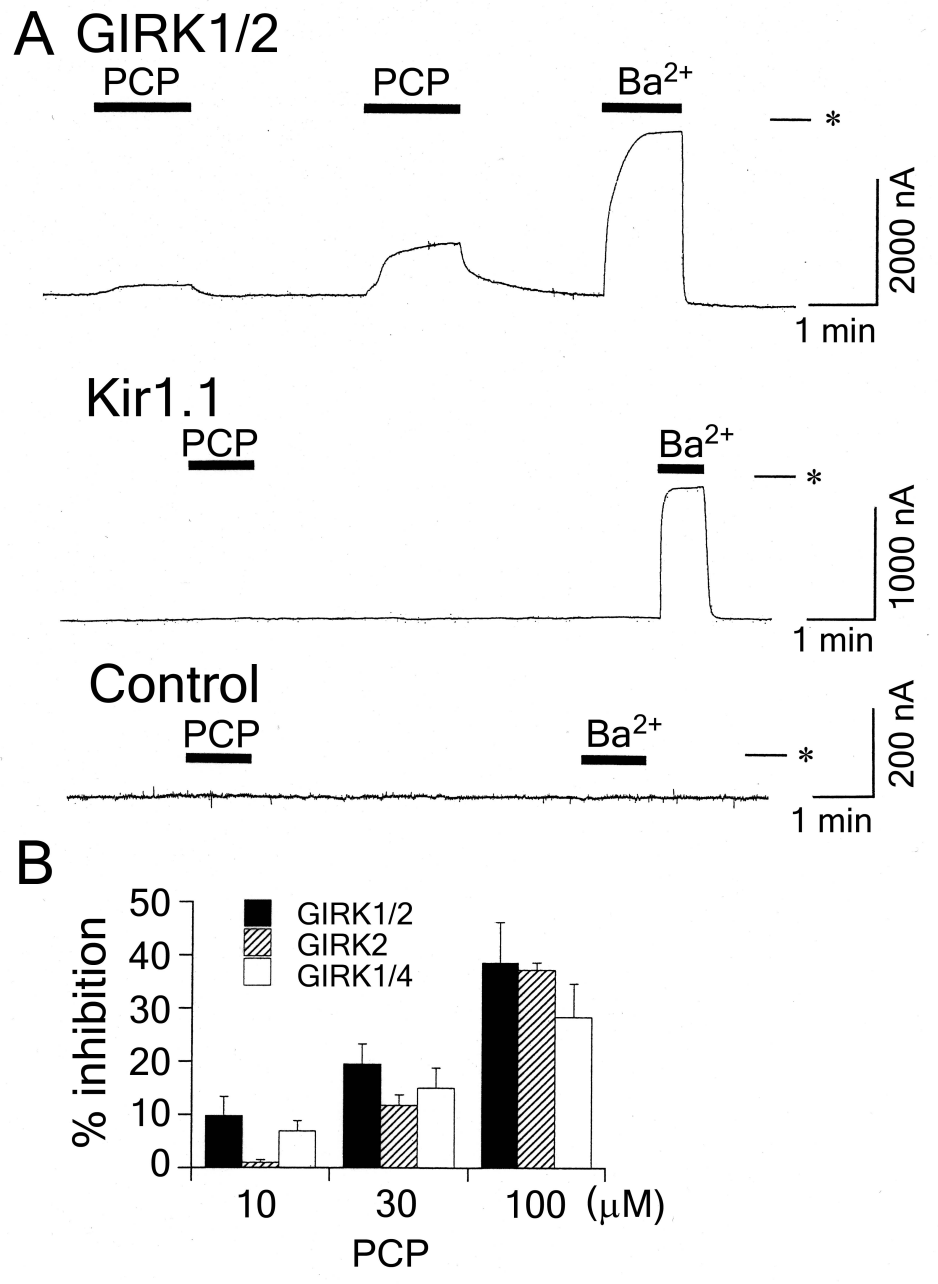
Inhibitory effects of PCP on GIRK channels expressed in *Xenopus* oocytes. (**A**) Top, in an oocyte injected with GIRK1 and GIRK2 mRNA, current responses to 10 µM and 100 µM PCP and to 3 mM Ba^2+^, a Kir channel blocker. Middle, in an oocyte injected with Kir1.1 mRNA, current responses to 100 µM PCP and to 3 mM Ba^2+^. Bottom, in an uninjected oocyte, no significant current responses to 100 µM PCP or 3 mM Ba^2+^. Asterisks show the zero current level. Bars show the duration of application. (**B**) Concentration-dependent inhibition of GIRK channels by PCP. The magnitudes of inhibition of GIRK currents by PCP were compared with the current components sensitive to 3 mM Ba^2+^.

**Fig. (2):**
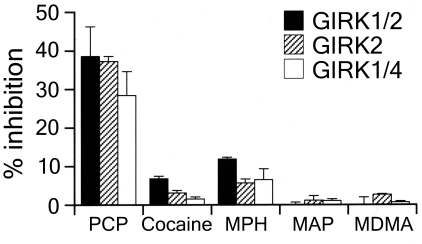
Comparison of the effects of five addictive drugs: PCP, cocaine, methylphenidate (MPH), methamphetamine (MAP) and MDMA, on GIRK channels. Drug concentration was 100 µM. The magnitudes of inhibition of GIRK currents by the drugs were compared with the 3 mM Ba^2+^-sensitive current components. Data except for PCP are from our previous study [12].
